# Supplementary material for: Development of Ex Vivo Analysis for Examining Cell Composition, Immunological Landscape, Tumor and Immune Related Markers in Non-Small-Cell Lung Cancer
Source: Cancers (Basel). 2024 Aug 20;16(16):2886. doi: 10.3390/cancers16162886 (PMC11352364; doi:10.3390/cancers16162886)
Supplement: Supplementary file 1 [file cancers-16-02886-s001.zip › cancers-3059950-SI.pdf]

Supplementary Materials: Development of Ex Vivo Analysis for Examining Cell Composition, Immunological Landscape, Tumor and Immune Related Markers in Non-Small-Cell Lung Cancer

Elena G. Ufimtseva, Margarita S. Gileva, Ruslan V. Kostenko, Vadim V. Kozlov and Lyudmila F. Gulyaeva

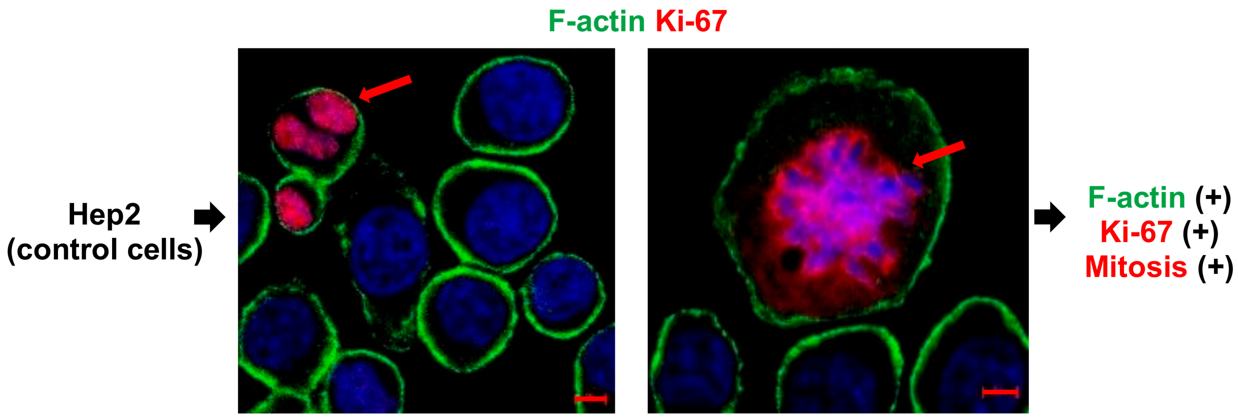

Figure S1. Representative confocal merged immunofluorescent images demonstrate the expression of the proliferation marker Ki-67 in Hep2 cells in *in vitro* culture as a positive control to Ki-67 staining. Cells and their nuclei are stained with the specific antibody to Ki-67 (green signal), TRITC-labeled phalloidin dye (red signal), and DAPI (blue signal), respectively. The magenta signal indicates some markers in the nuclei. Red arrows indicate mitosis. The scale bars are 5 µm each.

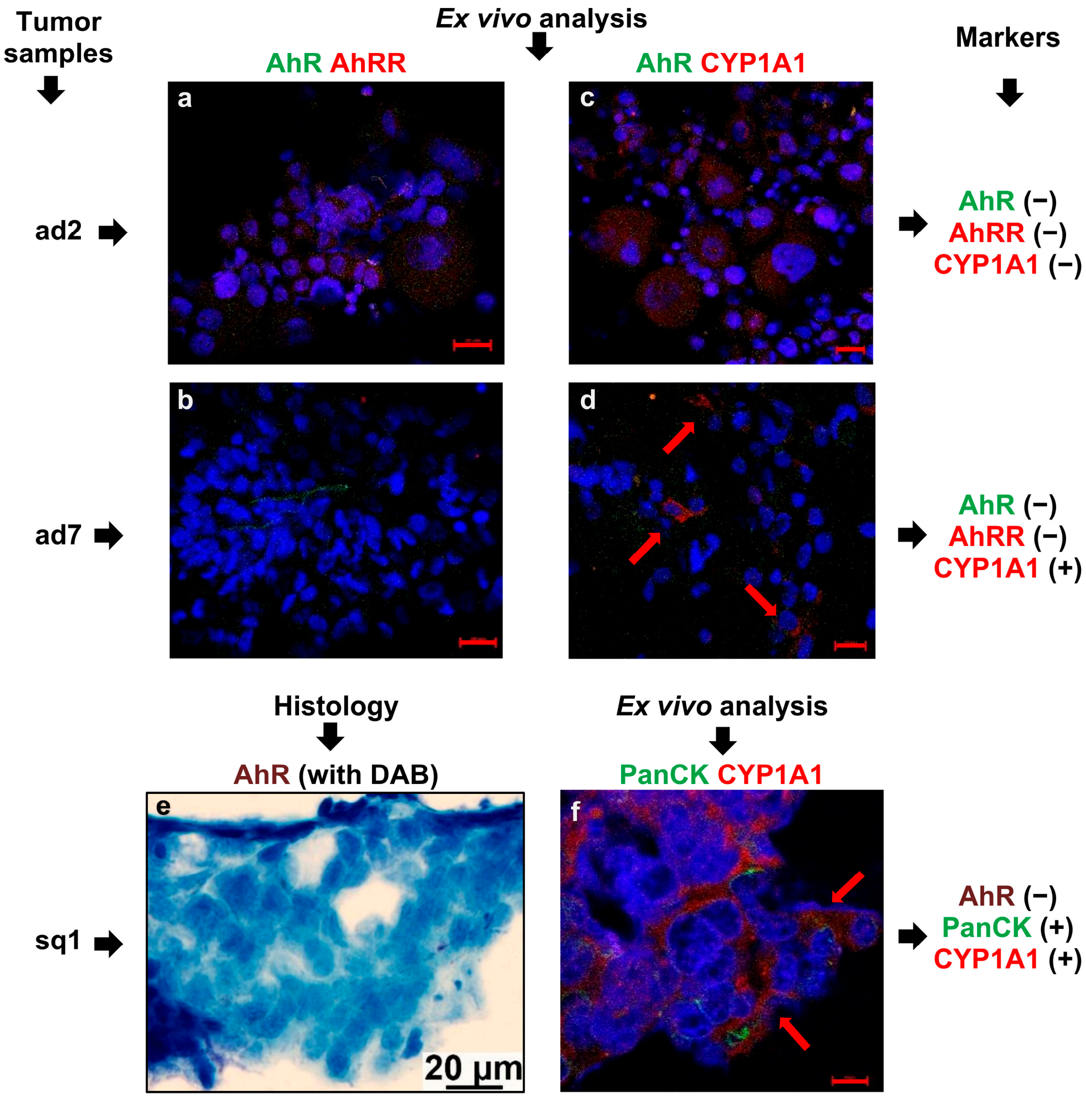

Figure S2. Representative confocal merged immunofluorescent and immunochemical images demonstrate that the cancer cells are not expressing the lung carcinogenesis markers AhR and AhRR on the *ex vivo* cell preparations and the histological section obtained from the same tumor samples of tobacco-smoking (tumor samples ad2 and sq1) and non-smoking (tumor sample ad7) patients, while the cancer cells obtained from some tumor samples do express the marker CYP1A1. Cells and their nuclei are stained with appropriate specific antibodies (green and red signals or brown staining) and DAPI (blue signal), respectively. Red arrows indicate the CYP1A1-positive cancer cells, as solitary and in clusters. The scale bars are (**f**) 10 and (**a**-**e**) 20 µm.

A

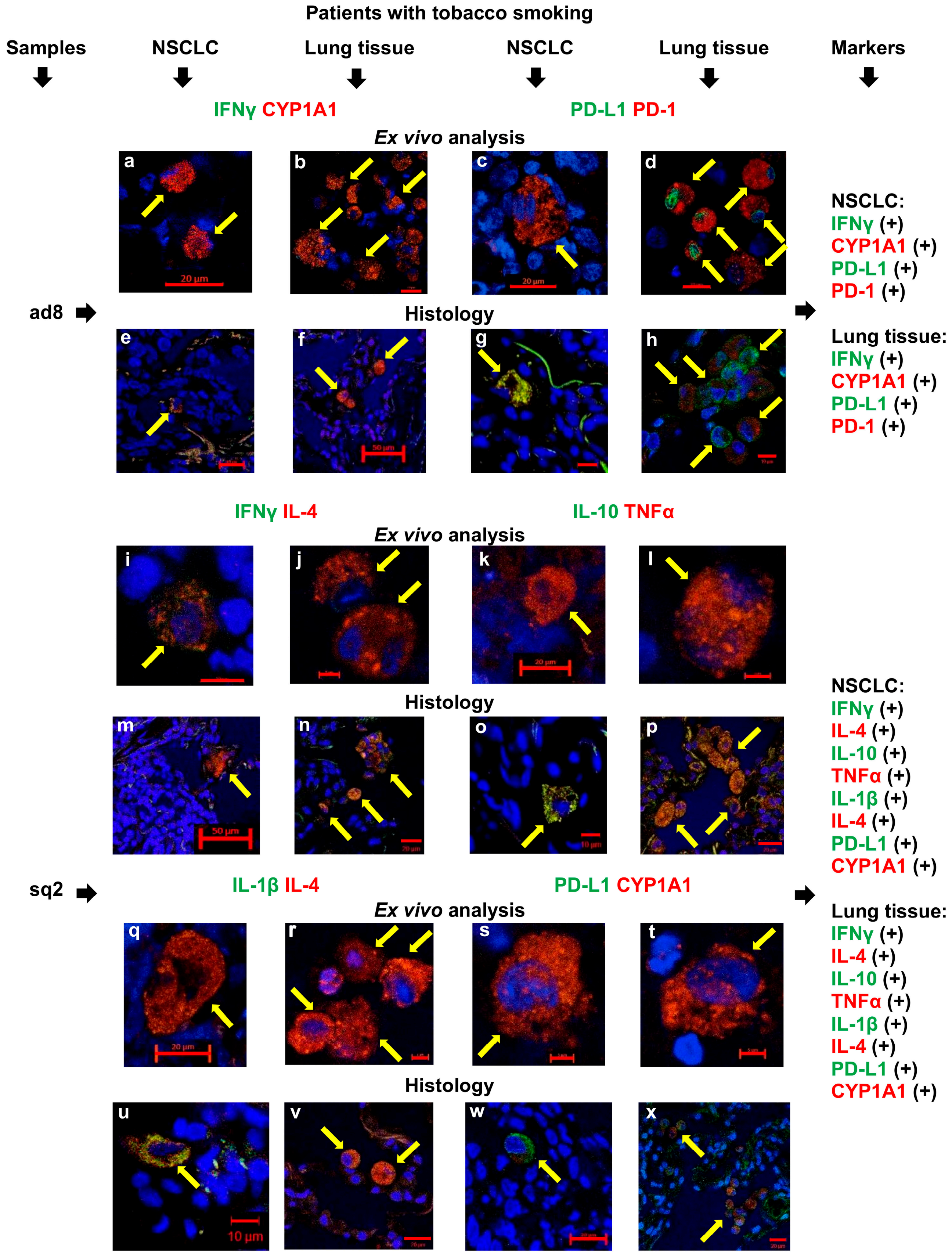
Patients with tobacco smoking
Samples
NSCLC
Lung tissue
NSCLC
Lung tissue
Markers
IFNγ CYP1A1
PD-L1 PD-1
Ex vivo analysis
a
b
c
d
20 µm
Histology
e
f
g
h
50 µm
ad8
NSCLC:
IFNγ (+)
CYP1A1 (+)
PD-L1 (+)
PD-1 (+)
Lung tissue:
IFNγ (+)
CYP1A1 (+)
PD-L1 (+)
PD-1 (+)
IFNγ IL-4
IL-10 TNFα
Ex vivo analysis
i
j
k
l
20 µm
Histology
m
n
o
p
50 µm
10 µm
sq2
IL-1β IL-4
PD-L1 CYP1A1
Ex vivo analysis
q
r
s
t
20 µm
Histology
u
v
w
x
10 µm
NSCLC:
IFNγ (+)
IL-4 (+)
IL-10 (+)
TNFα (+)
IL-1β (+)
IL-4 (+)
PD-L1 (+)
CYP1A1 (+)
Lung tissue:
IFNγ (+)
IL-4 (+)
IL-10 (+)
TNFα (+)
IL-1β (+)
IL-4 (+)
PD-L1 (+)
CYP1A1 (+)

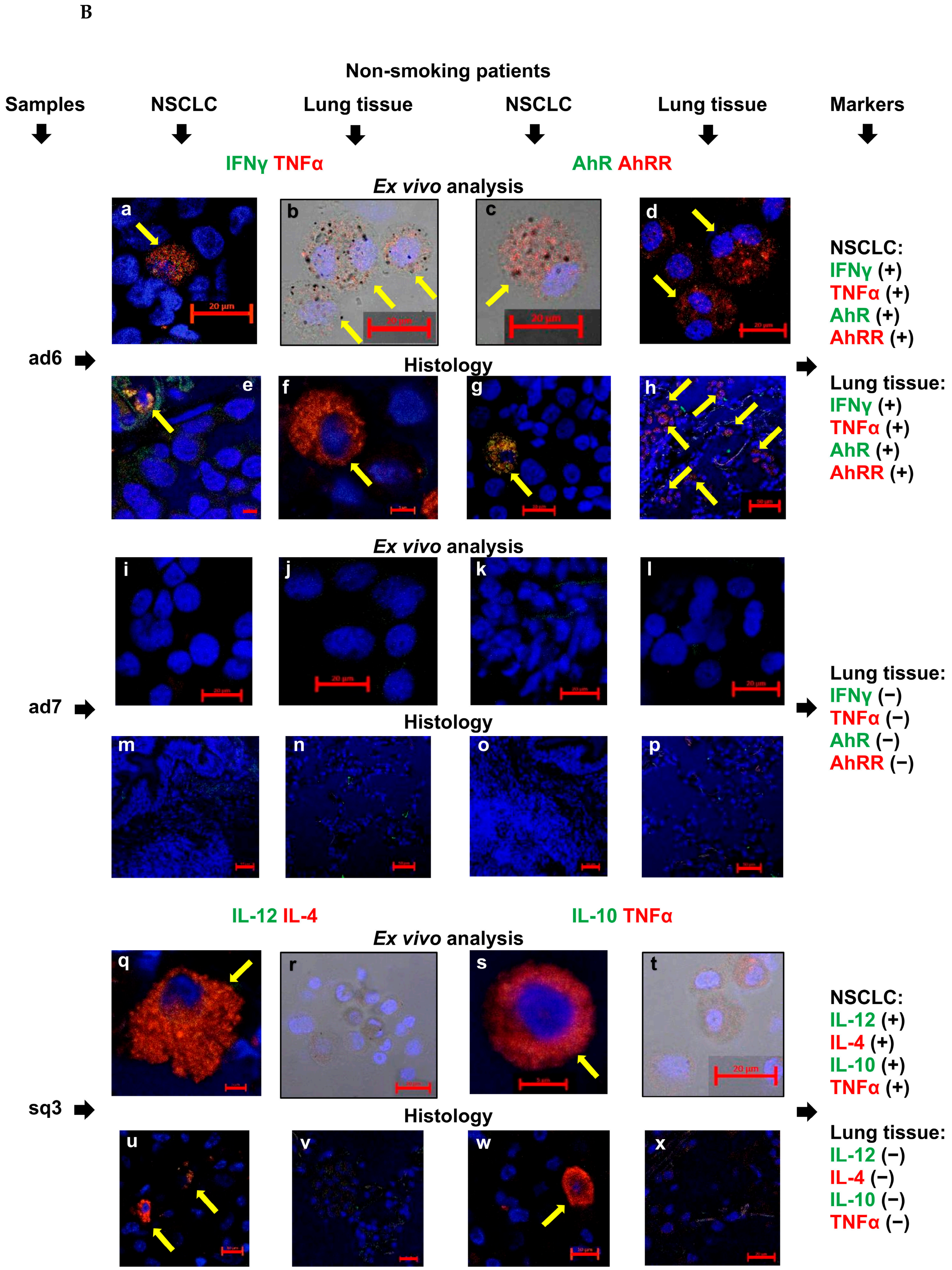

Figure S3. Representative confocal merged immunofluorescent images demonstrate the presence of immune markers expression in the most tumor-associated macrophages and alveolar macrophages

with denser dark inclusions in the cytoplasm obtained from the tumor and lung tissues, respectively, for (**A** – **a**-**x**) smoking and (**B** – **a**-**h**, **q**, **s**, **u, w**) non-smoking (according to medical records) patients, but (**B** – **i**, **l**, **n**, **p**, **r**, **t**, **v**, **x**) its absence in the alveolar macrophages without denser dark inclusions in the cytoplasm obtained from the lung tissues of some non-smoking patients on the *ex vivo* cell preparations and, in parallel, histological sections. Cells and their nuclei are stained with appropriate specific antibodies (green and red signals) and DAPI (blue signal), respectively. Colocalization of the markers is yellow signal. Yellow arrows indicate the markers-positive macrophages, as solitary and in clusters. (**B** – **b**, **c**, **r**, **t**) Phase-contrasted confocal immunofluorescent images are shown. The scale bars are (**A** – **j**, **e**, **r**-**t**; **B** – **e**, **f**, **q**, **s**) 5 µm, (**A** – **g**-**i**, **m**, **o**, **u**, **x**; **B** – **u**, **w**) 10 µm, (**A** – **a**-**e**, **k**, **n**, **p**, **q**, **v**, **w**; **B** – **a**-**d**, **g**, **i**-**e**, **r**, **t**, **v**, **x**) 20 µm, and (**A** – **f**; **B** – **h**, **m**-**p**) 50 µm.
